# Unrefined and Milled Ilmenite as a Cost-Effective Photocatalyst for UV-Assisted Destruction and Mineralization of PFAS

**DOI:** 10.3390/ma17153801

**Published:** 2024-08-01

**Authors:** Eustace Y. Fernando, Dibyendu Sarkar, Chatchai Rodwihok, Anshuman Satpathy, Jinxin Zhang, Roxana Rahmati, Rupali Datta, Christos Christodoulatos, Michel Boufadel, Steven Larson, Zhiming Zhang

**Affiliations:** 1 Department of Civil, Environmental and Ocean Engineering, Charles V. Schaefer, Jr. School of Engineering and Science, Rocco Technology Center, Stevens Institute of Technology, Hoboken, NJ 07030, USA; efernand3@stevens.edu (E.Y.F.); crodwiho@stevens.edu (C.R.); asatpath@stevens.edu (A.S.); rrahmati@stevens.edu (R.R.); christod@stevens.edu (C.C.); 2 Department of Chemistry and Chemical Biology, Charles V. Schaefer, Jr. School of Engineering and Science, Hoboken, NJ 07030, USA; jzhan50@stevens.edu; 3 Department of Biological Sciences, Michigan Technological University, Houghton, MI 49931, USA; rupdatta@mtu.edu; 4 Department of Civil and Environmental Engineering, New Jersey Institute of Technology, 323 MLK Blvd, Newark, NJ 07101, USA; boufadel@njit.edu; 5 U.S. Army Engineer Research and Development Center (ERDC), 3909 Halls Ferry Road, Vicksburg, MS 39180, USA; steven.l.larson@usace.army.mil; 6 Henry M. Rowan College of Engineering, Rowan University, Rowan Hall, 600 North Campus Drive, Glassboro, NJ 08028, USA; zhangz@rowan.edu

**Keywords:** raw ilmenite, photocatalyst, PFAS degradation, cost-effective catalyst, mineralization

## Abstract

Per- and polyfluoroalkyl substances (PFAS) are fluorinated and refractory pollutants that are ubiquitous in industrial wastewater. Photocatalytic destruction of such pollutants with catalysts such as TiO_2_ and ZnO is an attractive avenue for removal of PFAS, but refined forms of such photocatalysts are expensive. This study, for the first time, utilized milled unrefined raw mineral ilmenite, coupled to UV-C irradiation to achieve mineralization of the two model PFAS compounds perfluorooctanoic acid (PFOA) and perfluoro octane sulfonic acid (PFOS). Results obtained using a bench-scale photocatalytic reactor system demonstrated rapid removal kinetics of PFAS compounds (>90% removal in less than 10 h) in environmentally-relevant concentrations (200–1000 ppb). Raw ilmenite was reused over three consecutive degradation cycles of PFAS, retaining >80% removal efficiency. Analysis of degradation products indicated defluorination and the presence of shorter-chain PFAS intermediates in the initial samples. End samples indicated the disappearance of short-chain PFAS intermediates and further accumulation of fluoride ions, suggesting that original PFAS compounds underwent mineralization due to an oxygen-radical-based photocatalytic destruction mechanism induced by TiO_2_ present in ilmenite and UV irradiation. The outcome of this study implies that raw ilmenite coupled to UV-C is suitable for cost-effective reactor operation and efficient photocatalytic destruction of PFAS compounds.

## 1. Introduction

Contamination of surface and groundwater sources by per- and polyfluoroalkyl substances (PFAS) has become a concern in recent years due to their toxicity and refractory properties [1,2,3]. PFAS compounds comprise a highly hydrophobic fluorinated alkyl chain and a hydrophilic functional group at one edge. PFAS compounds are stable and unlikely to degrade in the environment due to the presence of strong C–F bonds in their structure [4]. Therefore, engineered treatment systems will be necessary for the reduction of environmental risks from this class of contaminants. Multiple techniques are being studied for the destruction of PFAS compounds, including thermal degradation [5], electrochemical oxidation [6], sonolysis [7], plasma destruction [7], supercritical water oxidation [8], and photocatalysis [9]. Photocatalytic destruction techniques are considered to be low-energy-consuming and sustainable, and they can be applied in ambient temperature [9]. In general, photocatalysis is an advanced oxidation process (AOP) that can oxidize a range of different organic compounds, including PFAS [10]. In this process, the target organic molecule absorbs a photon from a light source, and in the presence of a catalyst, the destruction of the organic molecule is accelerated [9]. 

Ilmenite is an (oxy)hydroxide mineral of iron and titanium with the same structure as that of hematite with two Fe^3+^ replaced by one Fe^2+^ and one Ti^4+^. The structural formula of the ilmenite is (Fe, Ti)_2_O_3_. Due to the presence of titanium dioxide in ilmenite’s structure, it is known to have photocatalytic properties [11]. Photocatalytic degradation of Reactive Black 5 dye was observed in the presence of natural ilmenite at an acidic pH upon irradiation with a 159 W xenon arc lamp (wavelength range 200–2500 nm) [12]. The destruction of the Reactive Black 5 dye was carried out by the hydroxyl radical generated during the Fenton reaction triggered by the dissolution of the Fe^2+^ present in the ilmenite structure. This is evidenced by a higher rate of destruction of the dye material at a lower pH, as more Fe^2+^ is dissolved at a lower pH. In another study, partial destruction of phenol into carboxylic acid was observed in the presence of ilmenite [13]. Similarly, complete photocatalytic destruction of Orange II dye was evidenced in the presence of ilmenite in a previous study [14]. These researchers also suggested that the Fenton reaction occurred, involving the dissolved structural Fe^2+^ as the primary mechanism of destruction of Orange II dye molecules. No photocatalytic degradation of any PFAS compound by ilmenite has yet been studied. 

Perfluorooctanoic acid (PFOA) and perfluoro octane sulfonic acid (PFOS) are the two abundant PFAS compounds that are found in the natural surface and groundwater sources due to anthropogenic pollution. They have been widely studied as representative PFAS compounds for multiple different adsorption and destruction studies in the last decade. Under environmentally relevant pH and ionic strengths, both PFOA and PFOS occur in anionic form. Due to the presence of a hydrophilic carboxylic or sulfonic functional group, the PFOA and PFOS anionic species can bind onto the surface of various oxyhydroxide and clay minerals like alumina, boehmite, hematite, montmorillonite, and kaolinite [15,16,17]. Recently, the sorption of PFOA and PFOS on amorphous aluminum (oxy)hydroxide phases has also been studied [18]. Ilmenite, due to its structural similarity with hematite, will likely have similar adsorption affinity for PFOA and PFOS anionic species as the hematite. However, the pH_pzc_ of ilmenite is expected to be low at pH 4.5, [12] making it effective for PFOA and PFOS adsorption only at a pH < 4.5. The isoelectric point (IEP) was determined as 6.25, 5.4, and 4.2 for Ilmenite-K, Ilmenite-Q, and Ilmenite-F, respectively [19].

The recalcitrance of PFAS compounds is partly due to fact that the C–F bonds can have a bond dissociation energy of up to 544 kJ/mol. Photocatalytic degradation of PFAS compounds has been explored in recent years using catalyst materials such as refined ZnO, TiO_2_, Ga_2_O_3_, and In_2_O_3_ [20]. Their derivatives such as TiO_2_/Ga_2_O_3_ coupled to multi-walled carbon nanotubes (MWCNTs) were also explored for their photocatalytic ability to remove recalcitrant PFAS compounds [21]. Although all of the aforementioned materials demonstrated excellent photocatalytic properties in removing PFAS, all of these are expensive, refined, and highly processed materials. Therefore, the cost of application of any of these to any wastewater PFAS removal process is likely to be high.

Ilmenite is a mineral composed of iron titanium oxide (FeTiO_3_) and it has been demonstrated to have photocatalytic capability where it photodegraded aromatic pollutants such as phenol [22]. Unrefined ilmenite is a much cheaper alternative to using refined TiO_2_ and other processed photocatalyst materials for PFAS destruction. 

Only a relatively small number of recent studies have reported the photocatalytic degradation of various PFAS compounds using various photocatalysts containing TiO_2_ [20,21,23,24]. All of these studies utilizing TiO_2_ as a photocatalyst have used highly refined or chemically modified forms of TiO_2_. Hitherto, there have been no reports of the use of a raw and unrefined mineral containing TiO_2_ for PFAS photocatalytic degradation. Therefore, the objective of this study was to use milled and raw ilmenite mineral sand as a cost-effective photocatalyst for UV-C irradiated photocatalytic destruction of PFOA and PFOS in a laboratory-scale reactor. 

## 2. Materials and Methods

### 2.1. Chemicals and Reagents

PFOA and PFOS used in this study were procured from Sigma Aldrich, St. Louis, MO, USA, and were of analytical grade. Ilmenite raw mineral in the milled and sieved state was procured from Euclid and PSH, ON, Canada. Supplier data indicated that the particle size of milled ilmenite ranged from 1.5 to 12 µm. 

### 2.2. Construction of the Photocatalytic Reactor and Photodegradation Experiments of PFOA and PFOS

**Figure 1:**
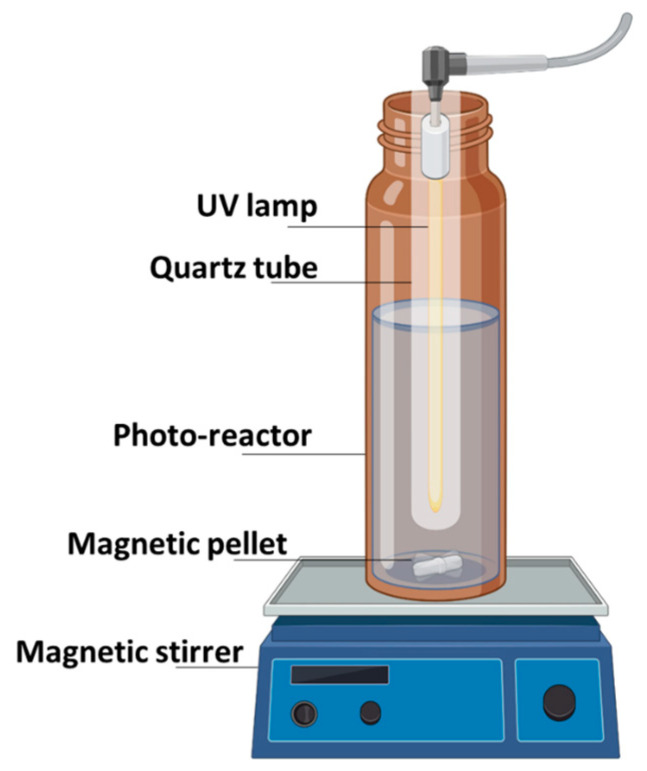
A schematic diagram of the ilmenite/UV-C photocatalytic reactor system used for degradation and mineralization of PFOA and PFOS.

The laboratory-scale custom-built cylindrical photocatalytic reactor composed of an amber borosilicate glass vessel with a magnetic stirring rod and an ultraviolet (UV) light source producing a continuous equivalent light intensity of 13 Watts, running down the central axis of the vessel, produced UV-C rays at 254 nm. The reactor was operated at room temperature (20 °C ± 2 °C), completely blocked off from external light sources during the experiments (Figure 1). For degradation kinetic experiments, the ilmenite was dosed at 400 mg/L. The pH was adjusted to 6.5 ± 0.5 during all experiments. PFOA and PFOS solutions of 200, 400, 600, 800, and 1000 ppb were prepared in 10 mM NaNO3 and were tested for photodegradation kinetics experiments. NaNO_3_ was added to the reaction medium to maintain a suitable and uniform ionic strength for the experiments. Samples were collected (5 mL) at 30 min intervals to track the temporal changes in the concentrations of the PFAS compounds. As control experiments, PFOA and PFOS solutions without the supplementation of ilmenite and another control that excluded all light sources inside the reaction vessel were used. Samples drawn at set intervals were filtered through 0.22 µm-pore-diameter regenerated cellulose (RC) filters (VWR, West Chester, PA, USA) before being analyzed for their PFAS contents by high-performance liquid chromatography–tandem mass spectrometry (HPLC–MS/MS). 

#### 2.2.1. Ilmenite Photocatalyst Reusability Study

To ascertain the reusability of the raw ilmenite photocatalyst for photodegradation of PFOA and PFOS in successive cycles of photocatalytic reactor operation, the system was operated for three consecutive cycles with PFOA and PFOS supplied at a concentration of 600 ppb, in separate experiments. The removal efficiency (RE %) of the two PFAS compounds was estimated (Equation (1)) and reported as a function of operation time to establish the potential reusability of the ilmenite photocatalyst material for successive cycles of non-stop operation.

(1) $$RE\left(\%\right)=\frac{\left(C_{0}-C\right)}{C_{0}}\times 100\;$$

#### 2.2.2. Model Fitting of Temporal Photocatalytic Degradation of PFOA and PFOS

The kinetic rates of photocatalytic activity of PFAS photocatalytic degradation were determined using pseudo-first-order kinetic models. (Equation (2)); *k* denotes the pseudo-first-order rate constant, C_0_ is the initial concentration of PFOA or PFOS, and C_t_ is the concentration at time “t”.

(2) $$ln\left(\frac{C_{t}}{C_{0}}\right)=-k\times t$$

Therefore, (−)*k* for the degradation of PFAS at each concentration would be equal to the slope of a linear plot of ln[C_t_/C_0_] against time (t).

### 2.3. Quantification of PFAS Compounds during and after Photocatalytic Degradation in Photocatalytic Reactor

The PFAS compounds were quantified throughout the PFOA and PFOS degradation kinetics experiments using the EPA standard method 1633 [25] by HPLC–MS/MS. Calibration plots for PFOA and PFOS by HPLC–MS/MS are shown in Figure S4A and Figure S4B, respectively. 

#### 2.3.1. HPLC Conditions and HPLC–MS/MS Quantification of PFAS

An Agilent 1100 HPLC system was coupled to a Micromass Quattro Ultima quadrupole mass spectrometer, controlled by MassLynx V4.0 software. The column oven temperature was maintained at 40 °C. An Xterra MS C18 analytical column (150 mm × 4.6 mm; 5.0 μm particle size, from Waters) and a Phenomenex delay column (50 mm × 4.6 mm; 3.0 μm particle size, from Phenomenex, Torrance, CA, USA) were used. The mobile phase consisted of aqueous ammonium acetate (20 mM) (solvent A) and acetonitrile (solvent B) and was pumped at a flow rate of 1.0 mL/min. The starting condition (10% B) was kept for 1 min. The proportion of B was increased linearly to 80% in 5.5 min and kept for 0.5 min. Subsequently, the mobile phase was adjusted to its initial composition in 1 min and held for 3.5 min for re-equilibration, resulting in a total run time of 11.5 min. Fifty microliters of each sample were injected into the column. The MS instrument was operated in the ESI negative mode and the data were acquired in multiple-reaction monitoring (MRM) mode. The MS tune parameters and compound parameters were optimized and determined with a syringe pump at a flow rate of 20 µL/min. The capillary voltage is 3.00 kV. The source temperature is 120 °C and the desolvation temperature is 300 °C. The cone voltage was 20.00 V for PFOA and 80.00 V for PFOS, respectively. The collision energy is 10.00 eV for PFOA and 50.00 eV for PFOS. 

#### 2.3.2. Analysis of Intermediate Products of PFOA and PFOS Mineralization by Ilmenite Photocatalytic Reactor

Shorter-chain intermediates of PFAS degradation of PFOA and PFOS were identified and measured using HPLC–MS/MS, as described in Section 2.3.1. Collected samples were prepared for HPLC–MS/MS analysis using PFAS-specific solid-phase extraction cartridges (Oasis WAX cartridges for PFAS, Waters Corporation, Milford, MA, USA). The resulting methanolic extracts were directly analyzed for their shorter-chain PFAS intermediates using the HPLC–MS/MS method, as described in Section 2.3.1.

### 2.4. Free Fluoride Measurements of PFOA and PFOS Photocatalytic Degradation Experiments

Fluoride release into the bulk liquid phase by defluorination of PFAS compounds during photocatalytic degradation experiments was measured. The samples were centrifuged at 4000 RPM, and the supernatant was filtered through a 0.22-micron-pore-diameter RC filter (VWR, Radnor, PA, USA). The filtered samples were diluted with an equal volume of TISAB II buffer solution (Thermo-Fisher Scientific, Waltham, MA, USA) and analyzed for their fluoride ion concentration using an Orion Star Meter equipped with the fluoride electrode (Thermo-Fisher Scientific, USA). The fluoride probe was calibrated using a fluoride ion calibration solution series between 5 µM and 90 µM. 

### 2.5. Physical and Chemical Characterization of the Photocatalyst Material

Characterization of the ilmenite photocatalyst was done using X-ray diffraction (XRD), scanning electron microscopy (SEM), and Brunauer–Emmett–Teller (BET) analysis. To gain the elemental and crystal structure information, ilmenite was characterized using a powder X-ray diffractometer (XRD, Rigaku SmartLab XRD, Auburn Hills, MI, USA). A nitrogen adsorption–desorption technique was used in conjunction with a Micromeritics ASAP 2020 PLUS Adsorption Analyzer (ASAP 2020 PLUS, Micromeritics, Norcross, GA, USA) to determine the specific surface area and pore volume, based on the Brunauer–Emmett–Teller (BET) equation.

### 2.6. Scanning Electron Microscopy (SEM) and Energy-Dispersive X-ray Spectroscopy (EDS) Analysis

Morphological and elemental analysis of the fresh and spent ilmenite photocatalyst was conducted using Zeiss AURIGA SEM equipped with an EDS analysis detector. Oxford’s INCA energy software (Version V1.08) was used to process data from the EDS analysis.

### 2.7. Fourier-Transform Infrared Spectroscopy (FTIR) of Fresh and Spent Ilmenite Photocatalyst Material

To surface-characterize the ilmenite photocatalyst material for chemical functional groups, FTIR spectra were obtained using an ATR-FTIR spectrometer (Bruker, Billerica, MA, USA) in the mid-IR wavenumber region (400–4000 cm^−1^), at a 2 cm^−1^ wavenumber resolution and 30 cumulative scans per sample. Tested samples included raw ilmenite mineral, recovered ilmenite during photocatalytic degradation of PFAS, and recovered ilmenite after photocatalytic degradation of PFAS. 

### 2.8. Radical Scavenger Experiments 

Several radical scavengers were tested for their efficacy in suppressing PFOA/PFOS degradation kinetic rates in the presence of raw ilmenite and UV-C irradiation, to obtain a mechanistic understanding of the photocatalytic PFAS degradation system. Four different scavengers (copper(II) nitrate, methanol, superoxide dismutase, and catalase) were tested to investigate the interference exerted into PFAS photodegradation by the ilmenite/UV-C system (Table 1). Cu(NO_3_)_2_ and methanol were exogenously introduced into the photocatalytic reactor vessel before UV-C irradiation, at a concentration of 5 mM, in the pH-adjusted (6.8 ± 0.2) reaction medium containing either PFOA or PFOS, at an initial concentration of 200 ppb. The two radical scavenging enzymes, superoxide dismutase (SOD) and catalase (Sigma Aldrich, St. Louis, MO, USA) (lyophilized at 2500 IU/mg protein) were supplemented at 2 IU final concentration into the photocatalytic reactor system from the stock solution (200 IU prepared in 50 mM pH-7 phosphate buffer). The control experiment did not contain any radical scavenging chemical species.

Samples were taken from the bulk solution at different time points, and they were analyzed for their remaining PFAS content by LC–MS, as described in Section 2.3.1. To calculate kinetic constants of photodegradation under the influence of radical scavenger chemical species, the PFAS concentrations were modeled using pseudo-first-order kinetic models as described in Section 2.2.2. 

## 3. Results and Discussion

### 3.1. Photocatalytic Degradation Kinetics of PFOA and PFOS

**Figure 2:**
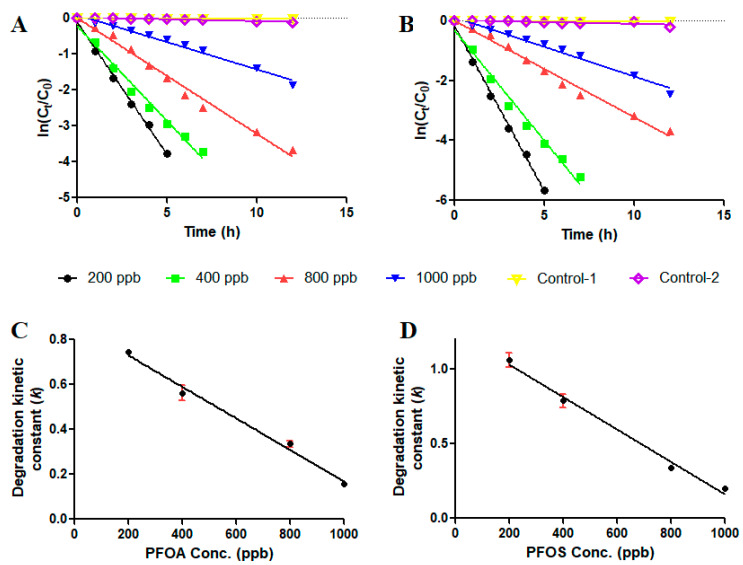
Photocatalytic degradation kinetics of PFOA and PFOS. Pseudo-first-order kinetic models of (**A**) PFOA and (**B**) PFOS removal and the variation of first-order kinetic constants as a function of (**C**) PFOA initial concentration and (**D**) PFOS initial concentration during photodegradation experiments with ilmenite as the photocatalyst material. Control-1 in both experiments was not ilmenite-supplemented but UV-irradiated, and Control-2 in both experiments had the UV lamp switched off. Both control experiments had a PFAS loading of 400 ppb.

Effective photocatalytic removal of both PFAS compounds was observed over a range of concentrations. Degradation of PFOA and PFOS at concentrations ranging from 200 to 1000 ppb was temporally tracked, and the ilmenite/UV-induced photocatalytic degradation kinetics of both compounds were modeled. The degradation kinetics of both compounds were best represented by pseudo-first-order kinetic models. A kinetic constant (k) was calculated for each PFAS degradation experiment, providing a direct comparison of removal kinetics of PFAS compounds supplemented at different concentrations. The highest k values (k = 0.8 h^−1^ for PFOA and 1.2 h^−1^ for PFOS) were observed for both PFAS compounds in the experiments where they were supplemented at the lowest concentration (200 ppb), indicating the most rapid removal rates. Both compounds were almost completely removed (>99%) from the bulk solution in under 5 h at this concentration (200 ppb) (Table 2, Figure 2A,B). Elevating the exogenously supplied PFAS content gradually degraded the pseudo-fist-order kinetic constant (k), indicating a slowing-down of the photocatalytic degradation rates of both PFOA and PFOS as PFOA and PFOS concentrations increased. The pseudo-first-order rate constant (k) for the photodegradation at 1000 ppb PFOA and PFOS solution slowed to 0.16 h^−1^ and 0.21 h^−1^, respectively (Table 2). Removal efficiency at the highest tested concentration of both PFOA and PFOS (1000 ppb) reached 98% after 24 h of continuous photocatalytic reactor operation.

The pseudo-first-order kinetic constants (*k*) of photocatalytic degradation indicated a linear decline as a function of their concentration for both compounds PFOA and PFOS (Figure 2C,D). This could be indicative of catalytic reaction sites of the raw ilmenite catalyst being saturated by high concentrations of PFAS pollutant molecules. Several previous studies utilizing TiO_2_ as a photocatalyst and UV indicated that model pollutants such as industrial dyes like Reactive Red-27 [26,27,28] and photocatalytic degradation of other pollutants such as p-nitrophenol by ZnO/UV [29] demonstrated that degradation kinetics of such pollutants by photocatalysis was best described using pseudo-first-order kinetic models. It was also demonstrated in such studies that increasing the initial concentration of pollutants produced a detrimental effect on pollutant degradation kinetic rates, leading to slower kinetic rates of photocatalytic degradation. 

From the control experiments (Figure 2A,B), it was evident that the contribution of adsorption of both PFAS compounds to the overall removal efficiency is less than 10% (Table 2) (7.7% for PFOA and 8.9% for PFOS). Therefore, the role of adsorption of PFAS compounds on the raw ilmenite catalyst material could be beneficial in initiating the initial contact between the two materials to trigger the photodegradation reaction in the presence of UV. The continuous removal of the adsorbed PFAS compounds on the raw ilmenite surfaces by photocatalysis is likely to expose occupied binding sites for PFAS binding, allowing the adsorption/degradation cycle to proceed. The limited contribution of adsorption to the overall removal percentage of both PFAS compounds studied in this work could also be explained by the relatively limited specific BET surface area and pore size reported for the raw ilmenite catalyst (11.144 m^2^∙g^−1^ and 8.031 nm) (Figure S1). The other control experiment (Control-1) demonstrated that the UV-C irradiation alone in the solutions containing PFAS was incapable of inducing a photocatalytic degradation reaction (Table 2). 

### 3.2. Reusability and Deterioration of the Ilmenite Photocatalyst Material over Successive Reaction Cycles 

**Figure 3:**
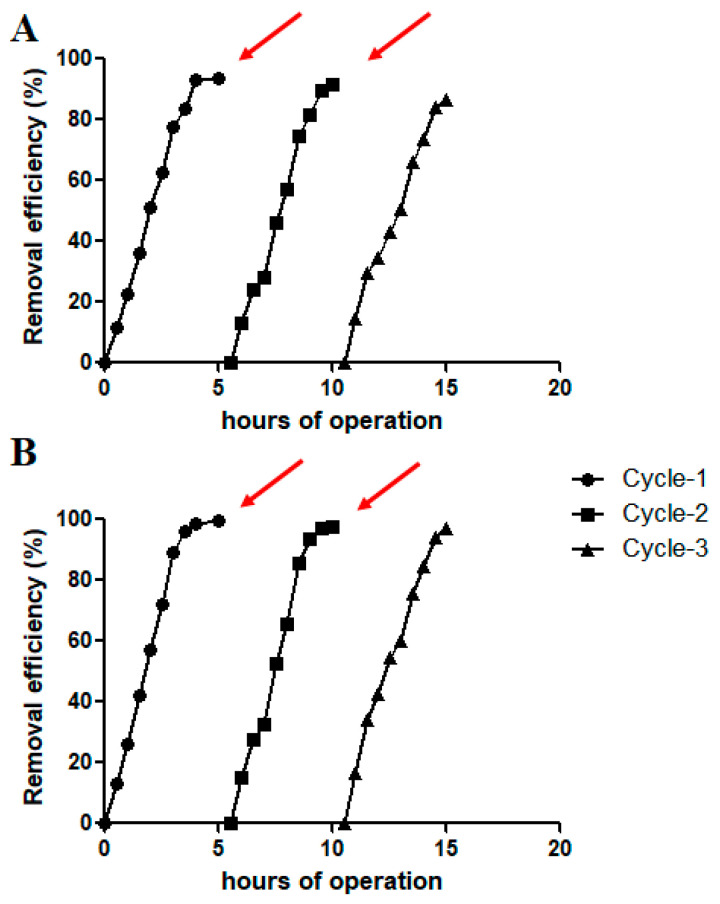
Reuse potential of the ilmenite photocatalyst. Photocatalytic degradation of (**A**) PFOA and (**B**) PFOS over three continuous operational cycles by ilmenite as the photocatalyst and a PFAS dosing of 600 ppb (raw ilmenite dosing = 800 mg/L). Arrows indicate PFAS supplementation into the photocatalytic reactor.

The reuse potential of the raw ilmenite photocatalyst material for successive cycles of UV-induced photodegradation reactions was studied over three successive treatment cycles. Moderately high PFAS dosing (600 ppb) was chosen for the experiments, and the PFAS removal in each cycle was expressed as a RE% for three successive cycles of operation. The results indicated that over three cycles of operation, the raw ilmenite photocatalyst indicated potential for reusability. Over 96% removal efficiency was observed for PFOS at the end of cycle-3, whereas over 86% removal efficiency was observed for PFOA in a similar experiment at the end of operational cycle-3 (Figure 3). 

The removal efficiency, however, dropped slightly over successive cycles, indicating a slight deterioration of the catalytic capacity of the raw ilmenite as a photocatalyst. For example, for PFOS, the RE% for the first and the third cycle were 99.8% and 96.1%, respectively. For PFOA, the RE% at the end of the first and the third operational cycles was 93.4% and 86.7%, respectively. Previous studies have also reported the reusability of TiO_2_/Fe_3_O_4_-based photocatalysts over several cycles of operation for methylene blue (MB) dye degradation [30]. The outcomes of the catalyst reusability study clearly demonstrated that the cost-effective raw ilmenite photocatalyst is capable of photodegradation of PFAS compounds over multiple successive reaction cycles.

### 3.3. Characterization of Raw Ilmenite 

#### 3.3.1. SEM and EDS Analysis of Ilmenite Photocatalyst Material

**Figure 4:**
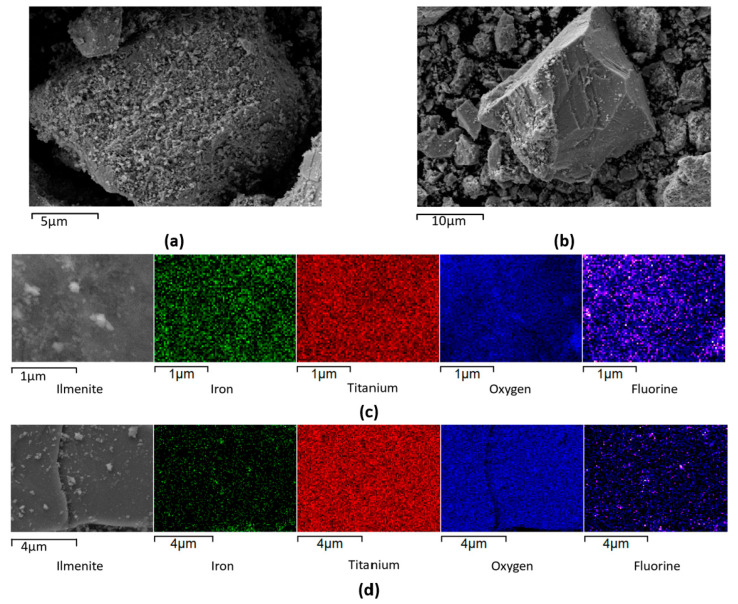
SEM image of ilmenite (**a**,**b**) and EDS mapping of ilmenite recovered from a PFOA solution before applying UV irradiation (**c**) and after UV irradiation (**d**).

The average particle size of the milled ilmenite particles ranged between 12 and 40 µm. SEM images of pristine ilmenite demonstrated particles with mostly porous surface structure (Figure 4a), which can indicate the weathering of ilmenite and oxidation of ferrous iron [31]. However, as Figure 4b indicates, some of the particles had a mostly flat, laminar surface with a flakey nature [32]. As Table 3 demonstrates, the ilmenite had some impurities (including silicon, carbon, and aluminum), which was expected for a natural mineral [33]; however, they made up less than 3–5% of the composition.

The PFOA and PFOS seem to associate with the ilmenite surface and appear to occur preferentially at the iron oxides/hydroxides on the surface of the ilmenite; as observed in Figure 4c,d, the amount of fluorine observed on the surface of the catalyst was lower when the iron concentration was lower, which can be another indication of the role of iron oxides in the adsorption of PFOA/PFOS. A loss of fluorine signal can be clearly observed in EDS maps after UV exposure, suggesting that the fluorine signal from adsorbed PFAS is largely diminished after it is exposed to UV irradiation, i.e., indication of adsorbed PFAS photodegradation. Elemental composition in terms of mass % and atomic % deduced using EDS elemental analysis indicated that raw ilmenite is mainly composed of Ti, Fe, and O. Carbon and silicon are present in trace amounts as impurities (Table 3).

#### 3.3.2. BET Analysis and Potential PFAS Adsorption by Raw Ilmenite Photocatalyst Material

To characterize the physical parameters related to the adsorption capacity of raw ilmenite samples based on the BET equation, an analysis of nitrogen adsorption–desorption isotherms was conducted as detailed in the Supplementary Data. Consequently, the specific surface area and pore size of the raw ilmenite samples were determined to be 11.144 m^2^∙g^−1^ and 8.031 nm (Figure S1), respectively. As seen in the nitrogen adsorption–desorption isotherm characteristic, this can be attributed to the presence of micro/nanopores on the surface morphology of ilmenite. Notably, the results of the BET analysis were found to be consistent with the corresponding SEM image. It is widely acknowledged that the surface area of ilmenite plays a crucial role in its adsorptive capacity, with micro/nanopores offering a greater number of active adsorption and photocatalytic sites. Thus, the presence of micro/nanopores on the surface of ilmenite can be considered a significant favorable characteristic for PFAS photodegradation. 

#### 3.3.3. FTIR Analysis of the Raw and Spent Ilmenite Photocatalyst after PFAS Degradation Experiments

FTIR analysis was performed of the raw ilmenite photocatalyst before it was utilized in the photodegradation experiments with PFAS compounds, before initiation of photocatalytic reactions by UV irradiation, and after complete degradation of PFAS compounds by photocatalysis. Raw ilmenite FTIR spectra from this study indicated the presence of characteristic O–Ti–O stretching vibration at 1058 cm^−1^ and Fe–O stretching vibrations at 1638 cm^−1^, and they are typical FTIR features found in raw ilmenite specimens (Figure S2) [34]. When ilmenite photocatalyst was recovered from the solutions containing PFAS, their FTIR spectrum indicated an additional peak occurring at 1277 cm^−1^, corresponding to –C–F stretching vibrations, indicating that the PFAS adsorbed on ilmenite photocatalyst is producing the additional FTIR feature corresponding to –C–F vibrations (Figure S2). However, when ilmenite photocatalyst was recovered after full depletion of PFAS in the bulk solution, the FTIR peak corresponding to –C–F stretching vibrations (1277 cm^−1^) was completely removed and the FTIR spectrum resembled raw ilmenite (Figure S2B). This demonstrates that adsorbed PFAS on ilmenite surfaces are mineralized and removed following UV irradiation. 

#### 3.3.4. XRD Analysis of Raw Ilmenite 

**Figure 5:**
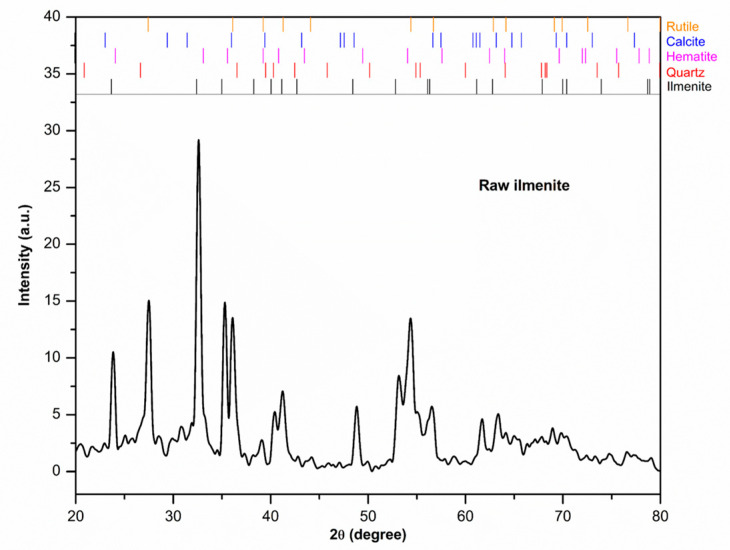
XRD pattern and identification result of the unrefined ilmenite.

The results obtained from X-ray diffraction (XRD) measurements suggest that the original ilmenite sample consists of various phases, as demonstrated by the qualitative analysis in Figure 5. These phases encompass ilmenite (FeTiO_3_; JCPDS No. 89-2811) [35], calcite (CaCO_3_; JCPDS No. 86-2340) [36], quartz (SiO_2_; JCPDS No. 46-1045) [18], hematite (Fe_2_O_3_; JCPDS No. 33-0664) [37], and rutile (TiO_2_; JCPDS No. 21-1276) [38]. The broadening and shift of the peak towards a higher 2θ degree could potentially be attributed to an increase in strain, lattice distortion, and the occurrence of impurities.

### 3.4. Defluorination and Mineralization of PFAS Compounds by Ilmenite/UV Photocatalysis

Potential degradation intermediates of PFAS photocatalytic degradation were studied using HPLC–MS/MS, and removal of fluoride ions into the bulk solution was also probed during the same experiments. Temporary formation of shorter-carbon-chain-length intermediates during the earlier time points of the experiments, as well as the release of fluoride into the bulk solution, was detected. 

#### 3.4.1. Defluorination of PFAS Compounds during Photocatalytic Degradation by UV/Ilmenite

**Figure 6:**
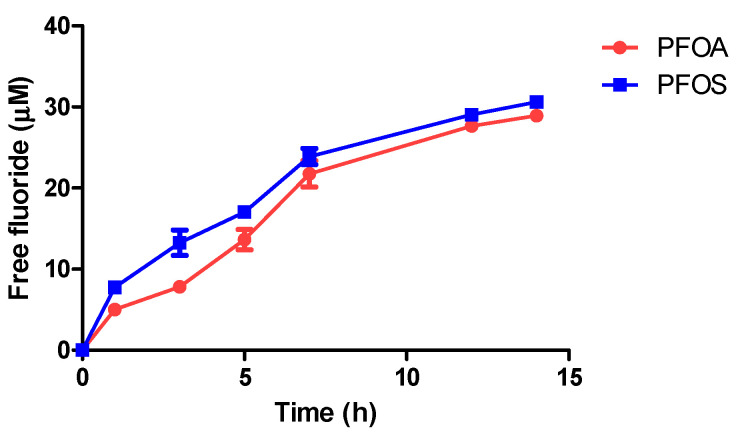
Release of fluoride ions into the bulk liquid medium during the photocatalytic degradation of PFOA and PFOS by UV/ilmenite (initial PFAS concentration in the experiment = 1000 ppb).

Free fluoride ions were detected in the bulk solutions of both PFOA and PFOS photodegradation experiments by UV/ilmenite photocatalytic system. Increasing amounts of free fluoride accumulation in the duration of the experiments were suggestive of dislodging of fluoride into the bulk solution by photocatalytic destruction/mineralization of PFAS molecules (Figure 6). 

Temporary formation and subsequent disappearance of shorter-chain PFAS compounds such as Perfluorobutanoic acid (PFBA) (four carbon) and perfluoropentanoic acid (PFPeA) (five carbon) were confirmed by HPLC–MS/MS analysis (Figure S3). The appearance of four- and five-carbon PFBA and PFPeA as intermediate compounds from the initial eight-carbon PFOA is indicative of oxidative reactions leading to chain shortening of PFOA, coupled with defluorination during the same process. The HPLC–MS/MS chromatograms of the end samples were completely devoid of any peaks that could be attributed to PFOA or its shorter-chain intermediates PFBA and PFPeA, observed earlier. This is a clear indication that the PFOA initially supplied underwent full mineralization with the raw ilmenite/UV photocatalytic system, leaving fluoride ions in the bulk liquid. 

#### 3.4.2. The Effect of Radical Scavenger Additions in Ilmenite and UV-C Photocatalytic Degradation of PFAS

**Figure 7:**
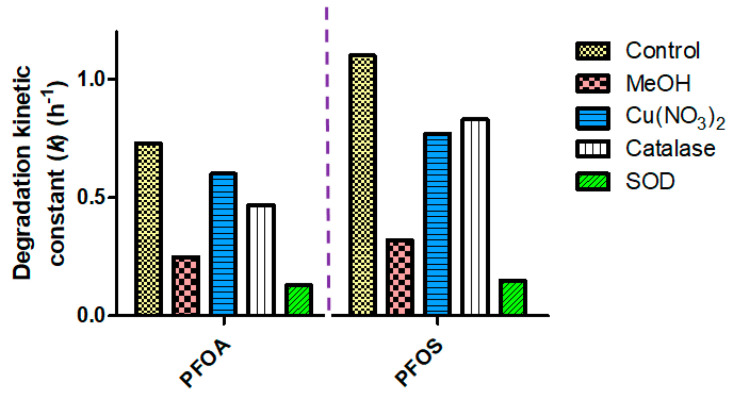
Effect of radical scavengers on photocatalysis. Addition of radical scavenger chemical species Cu(NO_3_)_2_, methanol, superoxide dismutase (SOD), and catalase altered the reaction rate of photocatalytic degradation of PFOA and PFOS in the tested ilmenite/UV-C reactor system.

The most pronounced detrimental effect to photocatalytic kinetic rates by the ilmenite/UV-C system for PFAS degradation was brought about by the supplementation of superoxide dismutase enzyme into the bulk medium. Compared to the control experiment (no scavenger), the kinetic constant rate (k) of PFAS degradation was suppressed 5.6-fold for PFOA and 7.3-fold for PFOS when SOD was added (Figure 7). SOD suppresses the superoxide anion radicals in solution and, therefore, this implies that superoxide anion radicals play a crucial role in the mechanism of PFAS degradation by unrefined ilmenite/UV-C system. 

The second greatest suppression of photocatalytic kinetic rates of PFAS degradation, compared to the control experiments, was brought about by the addition of methanol into the bulk reaction medium (a reduction of PFAS degradation kinetic constant (*k*) by 2.9-fold and 3.4-fold for PFOA and PFOS, respectively). Hydroxyl radicals are suppressed by the addition of methanol [39]. The least pronounced suppression of photocatalytic degradation of PFAS was brought about by the addition of catalase (suppresses H_2_O_2_) and Cu(NO_3_)_2_ (scavenges free electrons). These observations clearly demonstrate that the generation of superoxide anion radicals and hydroxyl radicals by the unrefined ilmenite/UV-C photocatalytic system is crucial for PFOA and PFOS mineralization. 

#### 3.4.3. Putative Photocatalytic Degradation Mechanisms of PFOA and PFOS by UV/Ilmenite Photocatalysis

**Figure 8:**
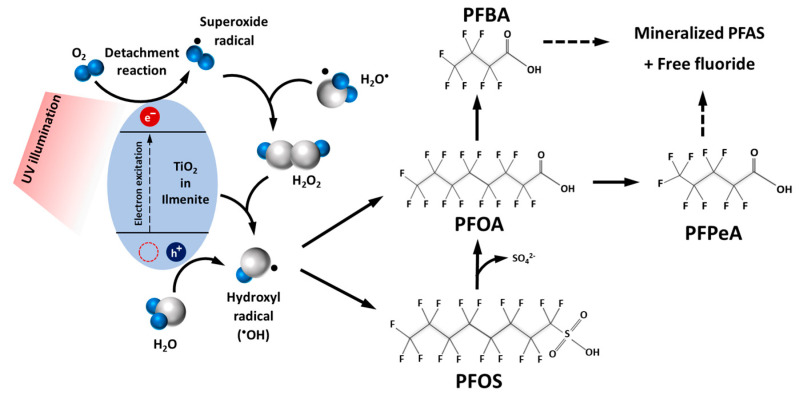
The putative photocatalytic degradation reaction schema of PFAS compounds by the UV/ilmenite photocatalytic system in this study.

The putative mechanism of PFAS mineralization by the UV/ilmenite photocatalytic degradation system appears to be proceeding through oxidative mechanisms driven by free-radical generation by the TiO_2_ contained in raw ilmenite mineral. Illumination of UV light could activate dissolved oxygen to produce superoxide anion radical [•O_2_^−^] in the presence of catalyst materials such as TiO_2_ and ZnO [19]. According to the putative mechanism proposed (Figure 8), excitation occurs when UV photons are present, causing electrons to move from the valence band to the conduction band. Subsequently, electrons and holes react with water molecules to generate active radicals. Recombination of electrons and holes also occurs during the photoreaction. When illuminated by UV, TiO_2_ contained within raw ilmenite could generate and excite an electron from the valence band [VB] to the conduction band [CB]. The electrons elevated to the conduction band interact with oxygen molecules, resulting in the formation of superoxide anion radicals [•O_2_^−^]. Through protonation, these radicals then transform into hydroperoxyl radicals [•HO_2_]. Subsequently, these radicals combine with captured electrons, giving rise to hydrogen peroxide [H_2_O_2_] and hydroxide radicals [•OH]. The formation of highly reactive radical oxygen species and hydrogen peroxide may lead to the initial production of shorter-chain cleavage products of four-carbon PFBA and five-carbon PFPeA, observed in the initial samples by HPLC–MS/MS (Figure S3). Earlier studies utilizing biosynthesized TiO_2_/UV [24] and mixed-metal oxide TiO2/UV [40] have proposed similar reaction mechanisms of photocatalytic degradation of PFAS compounds. The eventual disappearance of these intermediates in the end samples and accumulation of fluoride ions in the bulk solution are clearly indicative of subsequent oxidative destruction and mineralization of these intermediate PFAS breakdown products with prolonged exposure to the UV/ilmenite photocatalytic reactor system. 

The overall mechanism of PFAS capture and destruction by the UV/ilmenite photocatalytic system in this study can therefore be summarized as initial limited adsorption of PFAS on raw ilmenite surfaces, photoactivation of dissolved molecular oxygen on the catalysis surface by UV light, production of oxygen radicals with the aid of the TiO_2_ contained in raw ilmenite mineral, and complete mineralization of PFAS compounds via shorter-chain PFAS intermediates such as PFBA and PFPeA. 

This study utilized milled unrefined raw mineral ilmenite, coupled with UV-C irradiation, to achieve photocatalytic degradation and mineralization of the two model PFAS compounds PFOA and PFOS for the first time. The outcome of this work implies that cost-effective photocatalyst materials such as raw mineral ilmenite can be successfully utilized to fully mineralize PFAS compounds. It further suggests that raw ilmenite could potentially be used as a practical catalyst material for up-scaled photocatalytic reactor systems treating large volumes of PFAS-contaminated water. 

## 4. Conclusions

This study demonstrated that PFAS compounds at environmentally relevant concentrations (200–1000 ppb) can be effectively removed from aqueous media by the use of energy-efficient production of UV-C light and a cost-effective catalyst-material–raw-ilmenite-mineral in a simple photocatalytic reactor. Near-complete removal of model PFAS compounds PFOA and PFOS from the aqueous phase within acceptable UV irradiation timescales (less than 5 and 10 h at 200 and 1000 ppb, respectively, to achieve RE% > 90%) implies that the photocatalytic system is suitable for potential scaled-up operation to achieve acceptable levels of PFAS removal at a lower operational cost. The results of the study demonstrated that the raw ilmenite photocatalyst material is suitable for repeated rounds of photocatalytic destruction of PFAS compounds, with a minimum level of deterioration of its photocatalytic abilities. Degradation intermediates were identified and fluoride ion accumulation in the bulk medium was observed. This demonstrated that the PFAS compounds undergo mineralization via shorter-carbon-chain PFAS intermediates. Using raw ilmenite milled mineral sand for PFAS degradation offers a cost-effective way to effectively deal with highly fluorinated and refractory pollutants.

## Figures and Tables

**Table 1:** Radical scavenger species used in this study and the type of radical targeted by each chemical species.

Compound/Enzyme	Scavenger Type
Copper (II) nitrate	e^−^ scavenger
Methanol	•OH scavenger
Superoxide dismutase	O_2_•^−^ scavenger
Catalase	H_2_O_2_ scavenger

**Table 2:** Removal efficiencies of PFOA and PFOS photocatalytic degradation by ilmenite and UV irradiation.

PFAS Concentration (ppb)	PFOA			PFOS		
	Pseudo-First-Order Rate Constant (k) (h^−1^)	Maximum Removal Efficiency (%)	Coefficient of Determination (R^2^)	Pseudo-First-Order Rate Constant (k) (h^−1^)	Maximum Removal Efficiency (%)	Coefficient of Determination (R^2^)
200	0.8	99.7	0.99	1.2	99.49	0.99
400	0.59	99.1	0.98	0.83	99.2	0.98
800	0.35	98.6	0.99	0.39	98.8	0.98
1000	0.16	97.1	0.98	0.21	97.3	0.98
Control-1	0.002	0.09	0.98	0.003	ND	0.96
Control-2	0.01	7.72	0.97	0.02	8.91	0.97

ND—Not Detected. Experimental solutions contained 200–1000 ppb PFAS. Controls contained 400 ppb PFAS. Control-1: minus ilmenite and Control-2: minus UV.

**Table 3:** EDS elemental analysis of ilmenite.

Element	Ti	Fe	O	Al	C	Si
Mass %	35.7	24.44	37.59	0.66	1.53	0.08
Atomic %	20.21	11.87	63.72	0.67	3.45	0.08

## Data Availability

All the data generated in this study are included in the manuscript and the Supplementary Materials.

## Supplementary Materials

The following supporting information can be downloaded at: https://www.mdpi.com/article/10.3390/ma17153801/s1, Figure S1: BET adsorption/desorption curves for milled raw ilmenite mineral. Figure S2: FTIR spectra of the ilmenite photocatalyst before and after coming into contact with PFAS. Figure S3: LC chromatograms indicating the intermediate breakdown products formation during photocatalytic PFAS degradation. Figure S4: LC-MS/MS standard calibration curves of PFOA and PFOS.

## References

[1] Bolan N., Sarkar B., Yan Y., Li Q., Wijesekara H., Kannan K., Tsang D.C.W., Schauerte M., Bosch J., Noll H. (2021). Remediation of poly-and perfluoroalkyl substances (PFAS) contaminated soils–to mobilize or to immobilize or to degrade?. J. Hazard. Mater..

[2] Fenton S.E., Ducatman A., Boobis A., DeWitt J.C., Lau C., Ng C., Smith J.S., Roberts S.M. (2021). Per-and polyfluoroalkyl substance toxicity and human health review: Current state of knowledge and strategies for informing future research. Environ. Toxicol. Chem..

[3] Podder A., Sadmani A.H.M.A., Reinhart D., Chang N.-B., Goel R. (2021). Per and poly-fluoroalkyl substances (PFAS) as a contaminant of emerging concern in surface water: A transboundary review of their occurrences and toxicity effects. J. Hazard. Mater..

[4] Zhao L., Zhu L., Zhao S., Ma X. (2016). Sequestration and bioavailability of perfluoroalkyl acids (PFAAs) in soils: Implications for their underestimated risk. Sci. Total Environ..

[5] Alinezhad A., Challa Sasi P., Zhang P., Yao B., Kubátová A., Golovko S.A., Golovko M.Y., Xiao F. (2022). An investigation of thermal air degradation and pyrolysis of per-and polyfluoroalkyl substances and aqueous film-forming foams in soil. Acs Es&T Eng..

[6] Duinslaeger N., Radjenovic J. (2022). Electrochemical degradation of per-and polyfluoroalkyl substances (PFAS) using low-cost graphene sponge electrodes. Water Res..

[7] Sidnell T., Wood R.J., Hurst J., Lee J., Bussemaker M.J. (2022). Sonolysis of per-and poly fluoroalkyl substances (PFAS): A meta-analysis. Ultrason. Sonochem..

[8] McDonough J.T., Kirby J., Bellona C., Quinnan J.A., Welty N., Follin J., Liberty K. (2022). Validation of supercritical water oxidation to destroy perfluoroalkyl acids. Remediat. J..

[9] Meegoda J.N., Bezerra de Souza B., Casarini M.M., Kewalramani J.A. (2022). A review of PFAS destruction technologies. Int. J. Environ. Res. Public Health.

[10] Xia C., Lim X., Yang H., Goodson B.M., Liu J. (2022). Degradation of per-and polyfluoroalkyl substances (PFAS) in wastewater effluents by photocatalysis for water reuse. J. Water Process Eng..

[11] Chen D., Cheng Y., Zhou N., Chen P., Wang Y., Li K., Huo S., Cheng P., Peng P., Zhang R. (2020). Photocatalytic degradation of organic pollutants using TiO2-based photocatalysts: A review. J. Clean. Prod..

[12] Lee R.B., Lee K.M., Lai C.W., Pan G.-T., Yang T.C.K., Juan J.C. (2018). The relationship between iron and Ilmenite for photocatalyst degradation. Adv. Powder Technol..

[13] Moctezuma E., Zermeño B., Zarazua E., Torres-Martínez L.M., García R. (2011). Photocatalytic degradation of phenol with Fe-titania catalysts. Top. Catal..

[14] Pataquiva-Mateus A.Y., Zea H.R., Ramirez J.H. (2017). Degradation of Orange II by Fenton reaction using ilmenite as catalyst. Environ. Sci. Pollut. Res..

[15] Gao X., Chorover J. (2012). Adsorption of perfluorooctanoic acid and perfluorooctanesulfonic acid to iron oxide surfaces as studied by flow-through ATR-FTIR spectroscopy. Environ. Chem..

[16] Wang M., Orr A.A., Jakubowski J.M., Bird K.E., Casey C.M., Hearon S.E., Tamamis P., Phillips T.D. (2021). Enhanced adsorption of per-and polyfluoroalkyl substances (PFAS) by edible, nutrient-amended montmorillonite clays. Water Res..

[17] Zhao L., Bian J., Zhang Y., Zhu L., Liu Z. (2014). Comparison of the sorption behaviors and mechanisms of perfluorosulfonates and perfluorocarboxylic acids on three kinds of clay minerals. Chemosphere.

[18] Zhang Z., Sarkar D., Datta R., Deng Y. (2021). Adsorption of perfluorooctanoic acid (PFOA) and perfluorooctanesulfonic acid (PFOS) by aluminum-based drinking water treatment residuals. J. Hazard. Mater. Lett..

[19] Chavoshan S., Khodadadi M., Nasseh N. (2020). Photocatalytic degradation of penicillin G from simulated wastewater using the UV/ZnO process: Isotherm and kinetic study. J. Environ. Heal. Sci. Eng..

[20] Wu Y., Li Y., Fang C., Li C. (2019). Highly efficient degradation of perfluorooctanoic acid over a MnOx-modified oxygen-vacancy-rich In_2_O_3_ photocatalyst. ChemCatChem.

[21] Xu B. (2020). Photocatalysis of Aqueous Perfluorooctanoic Acid by TiO_2_ and Ga_2_O_3_ Assisted with Peroxymonosulfate under UV and Visible Light. Doctoral Dissertation.

[22] García-Muñoz P., Pliego G., Zazo J.A., Bahamonde A., Casas J.A. (2016). Ilmenite (FeTiO_3_) as low cost catalyst for advanced oxidation processes. J. Environ. Chem. Eng..

[23] Lashuk B., Pineda M., AbuBakr S., Boffito D., Yargeau V. (2022). Application of photocatalytic ozonation with a WO3/TiO2 catalyst for PFAS removal under UVA/visible light. Sci. Total Environ..

[24] Mustapha S., Tijani J.O., Elabor R., Etsuyankpa M.B., Amigun A.T., Shuaib D.T., Sumaila A., Olaoye A.J., Abubakar H.L., Abdulkareem S.A. (2023). Photocatalytic Degradation and Defluorination of Per- and Poly-Fluoroalkyl Substance (PFAS) Using Biosynthesized TiO_2_ Nanoparticles under UV-Visible Light. Eng. Proc..

[25] (2024). Analysis of Per- and Polyfluoroalkyl Substances (PFAS) in Aqueous, Solid, Biosolids, and Tissue Samples by LC-MS/MS.

[26] Daneshvar N., Rabbani M., Modirshahla N., Behnajady M.A. (2004). Kinetic modeling of photocatalytic degradation of Acid Red 27 in UV/TiO_2_ process. J. Photochem. Photobiol. A Chem..

[27] Mahmoodi N.M., Arami M., Limaee N.Y., Tabrizi N.S. (2006). Kinetics of heterogeneous photocatalytic degradation of reactive dyes in an immobilized TiO_2_ photocatalytic reactor. J. Colloid Interface Sci..

[28] Kiwaan H.A., Atwee T.M., Azab E.A., El-Bindary A.A. (2020). Photocatalytic degradation of organic dyes in the presence of nanostructured titanium dioxide. J. Mol. Struct..

[29] Kadam V.V., Shanmugam S.D., Ettiyappan J.P., Balakrishnan R.M. (2021). Photocatalytic degradation of p-nitrophenol using biologically synthesized ZnO nanoparticles. Environ. Sci. Pollut. Res..

[30] Tumbelaka R.M., Istiqomah N.I., Kato T., Oshima D., Suharyadi E. (2022). High reusability of green-synthesized Fe_3_O_4_/TiO_2_ photocatalyst nanoparticles for efficient degradation of methylene blue dye. Mater. Today Commun..

[31] Parirenyatwa S., Escudero-Castejon L., Sanchez-Segado S., Hara Y., Jha A. (2016). Comparative study of alkali roasting and leaching of chromite ores and titaniferous minerals. Hydrometallurgy.

[32] Al-Sabagh A.M., Abdou M.I., Migahed M.A., Fadl A.M., Farag A.A., Mohammedy M.M., Abd-Elwanees S., Deiab A. (2018). Influence of ilmenite ore particles as pigment on the anticorrosion and mechanical performance properties of polyamine cured epoxy for internal coating of gas transmission pipelines. Egypt. J. Pet..

[33] Pham X.N., Pham D.T., Ngo H.S., Nguyen M.B., Doan H. (2021). V Characterization and application of C–TiO_2_ doped cellulose acetate nanocomposite film for removal of Reactive Red-195. Chem. Eng. Commun..

[34] Wijewardhana T.D.U., Ratnayake A.S. (2021). Applicability of carbothermic reduction for upgrading Sri Lankan ilmenite ores: Towards converting ilmenite into synthetic rutile by mechanical activation. Bull. Natl. Res. Cent..

[35] Phoohinkong W., Pavasupree S., Wannagon A., Sanguanpak S., Boonyarattanakalin K., Mekprasart W., Pecharapa W. (2017). Characterization and x-ray absorption spectroscopy of ilmenite nanoparticles derived from natural ilmenite ore via acid-assisted mechanical ball-milling process. Adv. Nat. Sci. Nanosci. Nanotechnol..

[36] Pande G., Selvakumar S., Ciotonea C., Giraudon J.-M., Lamonier J.-F., Batra V.S. (2021). Modified red mud catalyst for volatile organic compounds oxidation. Catalysts.

[37] Hu S., Wu Y., Ding Z., Shi Z., Li F., Liu T. (2020). Facet-dependent reductive dissolution of hematite nanoparticles by Shewanella putrefaciens CN-32. Environ. Sci. Nano.

[38] Tang Z., Xu L., Shu K., Yang J., Tang H. (2022). Fabrication of TiO_2_@ MoS_2_ heterostructures with improved visible light photocatalytic activity. Colloids Surfaces A Physicochem. Eng. Asp..

[39] Pelaez M., Falaras P., Likodimos V., O’Shea K., de la Cruz A.A., Dunlop P.S.M., Byrne J.A., Dionysiou D.D. (2016). Use of selected scavengers for the determination of NF-TiO_2_ reactive oxygen species during the degradation of microcystin-LR under visible light irradiation. J. Mol. Catal. A Chem..

[40] Yang H., Park S.-J., Lee C.-G. (2024). Enhanced removal of perfluoroalkyl substances using MMO-TiO_2_ visible light photocatalyst. Alex. Eng. J..

