# Supplementary material for: Unrefined and Milled Ilmenite as a Cost-Effective Photocatalyst for UV-Assisted Destruction and Mineralization of PFAS
Source: Materials (Basel). 2024 Aug 1;17(15):3801. doi: 10.3390/ma17153801 (PMC11312862; doi:10.3390/ma17153801)
Supplement: Supplementary file 1 [file materials-17-03801-s001.zip › materials-3108769-supplementary.pdf]

Supplementary data

Unrefined and Milled Ilmenite as a Cost-Effective Photocatalyst for UV-assisted destruction and Mineralization of PFAS

Eustace Y. Fernando[1], Dibyendu Sarkar[1*], Chatchai Rodwihok[1], Anshuman Satpathy[1], Jinxin Zhang[2], Roxana Rahmati[1], Rupali Datta[3], Christos Christodoulatos[1], Michel Boufadel[4], Steven Larson[5], and Zhiming Zhang[6]

[1]Department of Civil, Environmental and Ocean Engineering, Charles V. Schaefer, Jr. School of Engineering and Science, Rocco Technology Center, Hoboken, NJ. 07030, USA

[2]Department of Chemistry and Chemical Biology, Charles V. Schaefer, Jr. School of Engineering and Science, Hoboken, NJ. 07030, USA;

[3]Department of Biological Sciences, Michigan Technological University, Houghton, MI 49931, USA;

[4] Department of Civil and Environmental Engineering, New Jersey Institute of Technology, 323 MLK Blvd, Newark, NJ 07101, USA;

[5]U.S. Army Engineer Research and Development Center (ERDC), 3909 Halls Ferry Road, Vicksburg, Mississippi, 39180. USA;

[6] Henry M. Rowan College of Engineering, Rowan Hall, 600 North Campus Drive, Glassboro, NJ 08028. USA;

* Correspondence: dsarkar@stevens.edu; Tel.: 201-216-8028

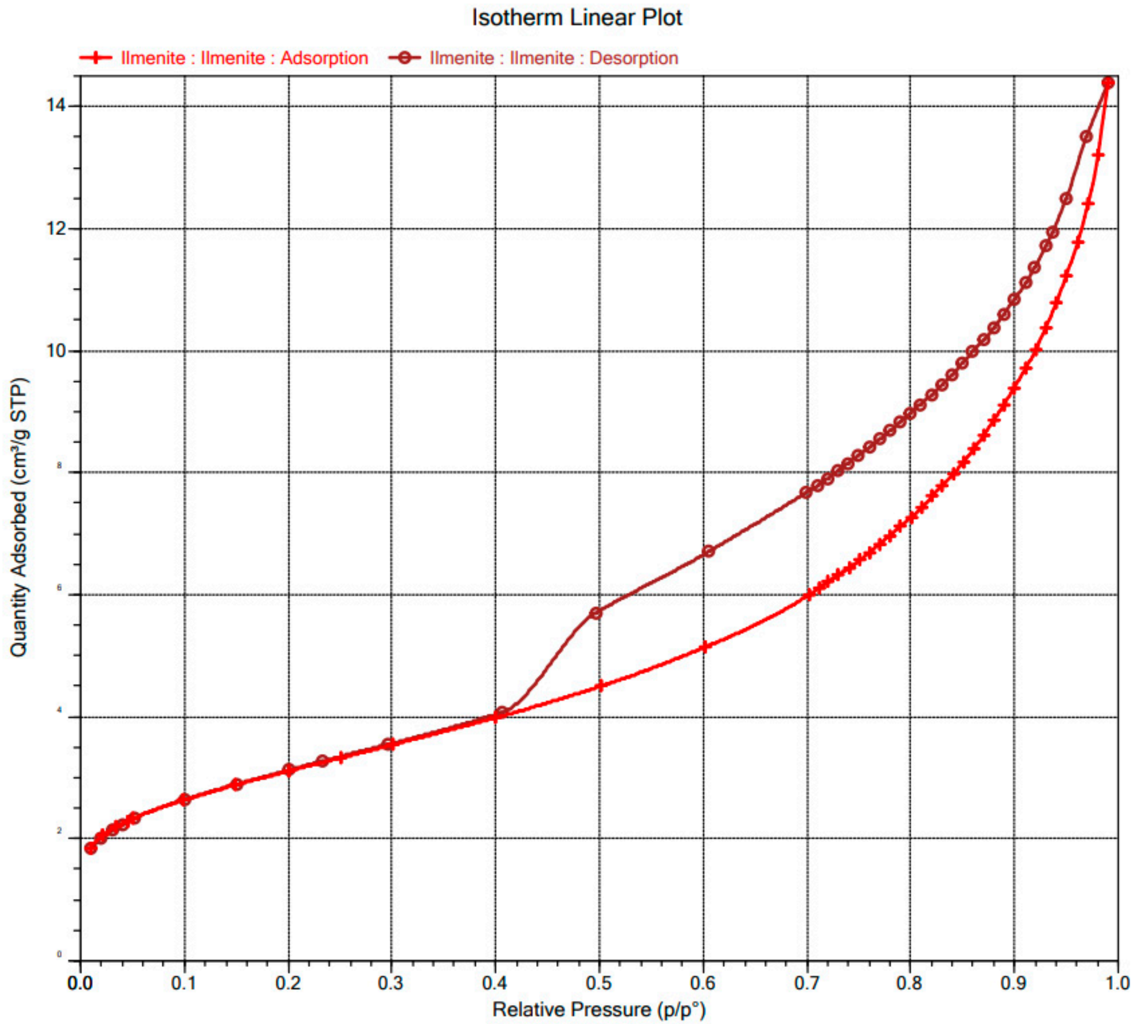

Figure S1: BET adsorption/desorption curves for milled raw ilmenite mineral indicated a specific surface area and pore size of the raw ilmenite samples were 11.144 m2·g−1 and 8.031 nm, respectively.

A

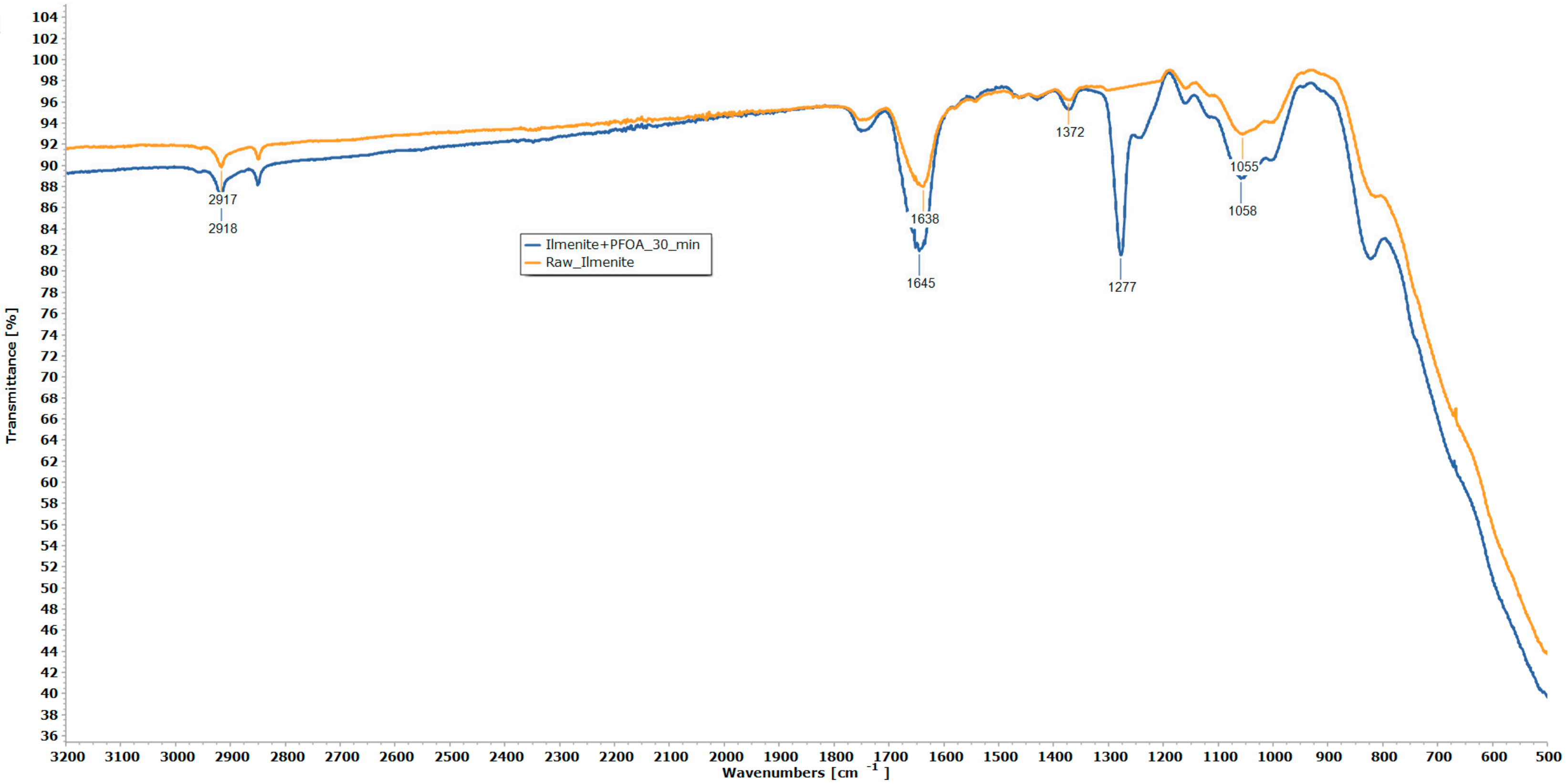

Transmittance [%]
Wavenumbers [cm -1]
Ilmenite+PFOA_30_min
Raw_Ilmenite
2917
2918
1638
1645
1372
1277
1055
1058

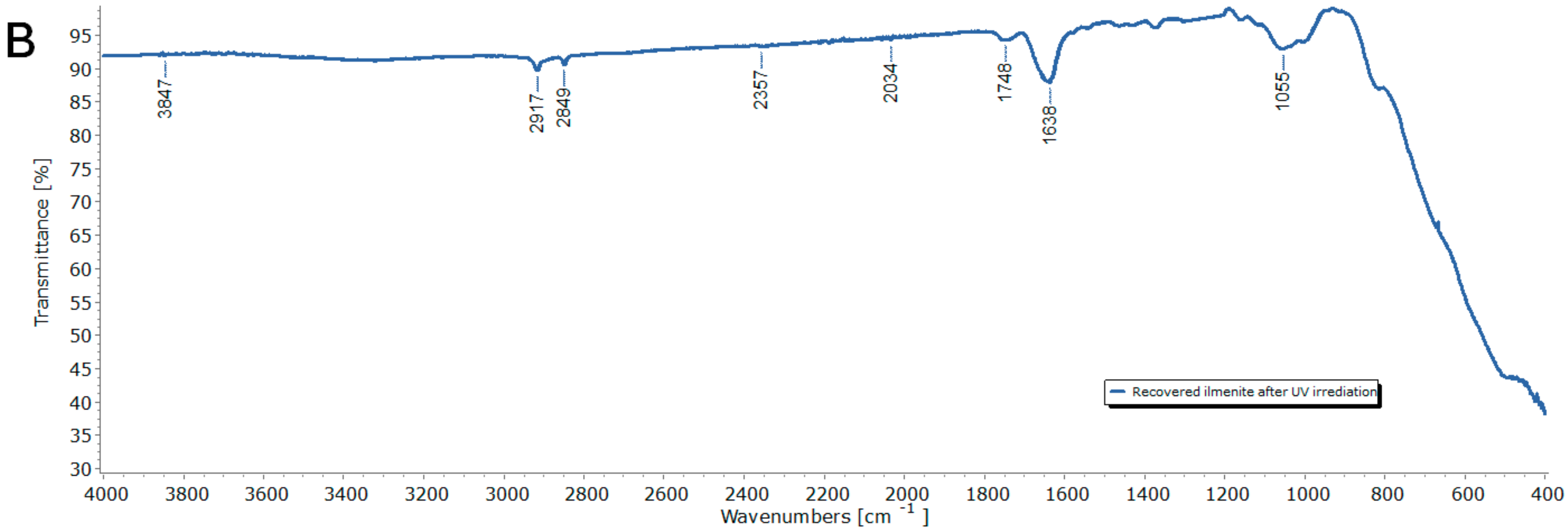

Figure S2: FTIR spectra of the (A) raw ilmenite photocatalyst (orange line) and recovered after complete degradation of PFAS compounds by photocatalysis (blue line). O-Ti-O stretching vibration at 1058 cm^{-1} and Fe-O stretching vibrations at 1638 cm^{-1} are common in all spectra, indicating the presence of unchanged raw ilmenite in all stages and the presence of additional –C-F stretching vibrational IR peak at 1277 cm^{-1} indicating adsorbed PFAS on ilmenite surfaces before undergoing photocatalytic degradation and (B) ilmenite photocatalyst recovered after UV irradiation and photocatalytic degradation experiments of PFAS.

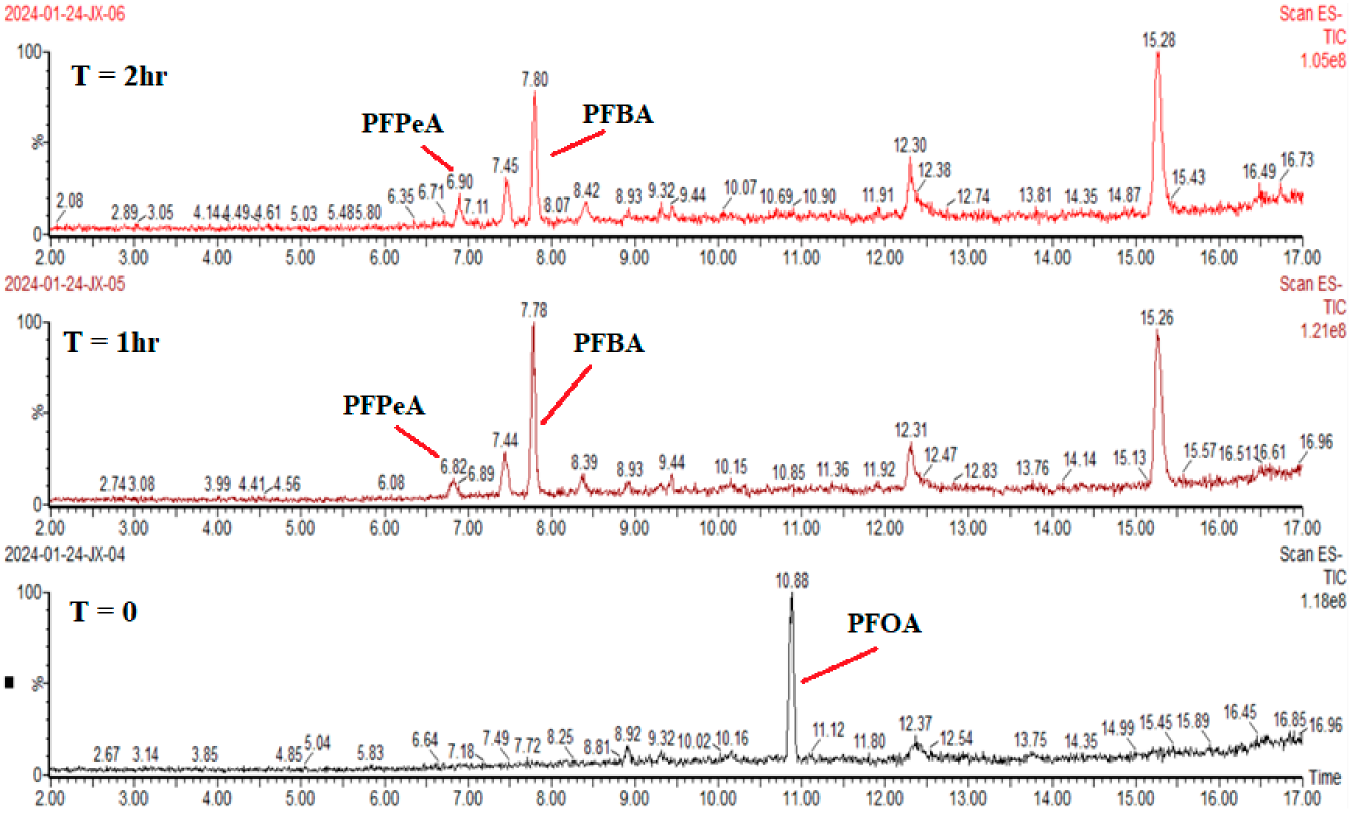

Figure S3: Representative UPLC chromatograms indicating the intermediate breakdown products formation during photocatalytic PFAS degradation experiments at different time points during PFAS photocatalytic degradation experiments (T=1 hr and T = 2 hrs compared to T=0 hr sample) indicates the presence of intermediate degradation products with shorter carbon lengths (PFBA and PFPeA).

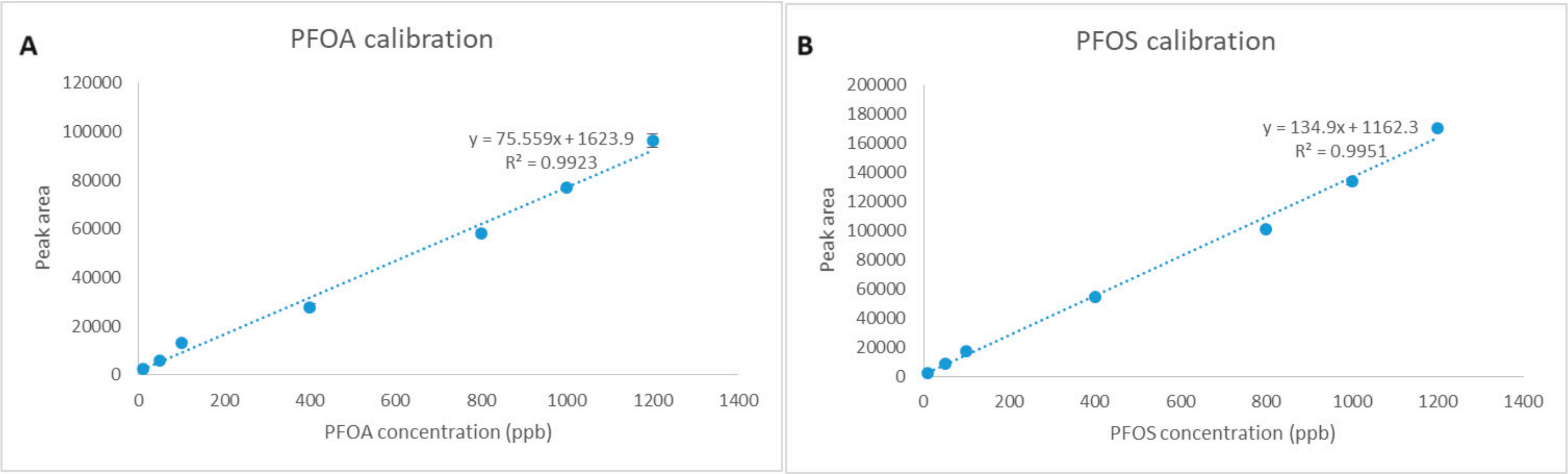

Figure S4: LC-MS/MS standard calibration curves of (A) PFOA and (B) PFOS. Limits of quantification (LOQs) for PFOA and PFOS with the instrument parameters were 1.2 ppt and 2 ppt, respectively.
